# Effective bioremediation strategy for rapid *in situ* cleanup of anoxic marine sediments in mesocosm oil spill simulation

**DOI:** 10.3389/fmicb.2014.00162

**Published:** 2014-04-14

**Authors:** Maria Genovese, Francesca Crisafi, Renata Denaro, Simone Cappello, Daniela Russo, Rosario Calogero, Santina Santisi, Maurizio Catalfamo, Alfonso Modica, Francesco Smedile, Lucrezia Genovese, Peter N. Golyshin, Laura Giuliano, Michail M. Yakimov

**Affiliations:** ^1^Institute for Coastal Marine Environment, CNRMessina, Italy; ^2^Department of Biological and Environmental Sciences, University of MessinaMessina, Italy; ^3^Environmental Laboratory, Syndial SpAPriolo Gargallo, Italy; ^4^Environmental Genomics, School of Biological Sciences, Bangor UniversityBangor, UK; ^5^Mediterranean Science CommissionMonaco, Monaco

**Keywords:** marine anoxic sediments, crude oil pollution, hydrocarbonoclastic bacteria, *in situ* bioremediation, aerated slurry system

## Abstract

The purpose of present study was the simulation of an oil spill accompanied by burial of significant amount of petroleum hydrocarbons (PHs) in coastal sediments. Approximately 1000 kg of sediments collected in Messina harbor were spiked with Bunker C furnace fuel oil (6500 ppm). The rapid consumption of oxygen by aerobic heterotrophs created highly reduced conditions in the sediments with subsequent recession of biodegradation rates. As follows, after 3 months of ageing, the anaerobic sediments did not exhibit any significant levels of biodegradation and more than 80% of added Bunker C fuel oil remained buried. Anaerobic microbial community exhibited a strong enrichment in sulfate-reducing PHs-degrading and PHs-associated *Deltaproteobacteria*. As an effective bioremediation strategy to clean up these contaminated sediments, we applied a Modular Slurry System (MSS) allowing the containment of sediments and their physical–chemical treatment, e.g., aeration. Aeration for 3 months has increased the removal of main PHs contaminants up to 98%. As revealed by CARD-FISH, qPCR, and 16S rRNA gene clone library analyses, addition of Bunker C fuel oil initially affected the activity of autochthonous aerobic obligate marine hydrocarbonoclastic bacteria (OMHCB), and after 1 month more than the third of microbial population was represented by *Alcanivorax-, Cycloclasticus*-, and *Marinobacter*-related organisms. In the end of the experiment, the microbial community composition has returned to a status typically observed in pristine marine ecosystems with no detectable OMHCB present. Eco-toxicological bioassay revealed that the toxicity of sediments after treatment was substantially decreased. Thus, our studies demonstrated that petroleum-contaminated anaerobic marine sediments could efficiently be cleaned through an *in situ* oxygenation which stimulates their self-cleaning potential due to reawakening of allochtonous aerobic OMHCB.

## Introduction

The worldwide production of crude oil and natural gas is at the peak, with an estimated worldwide production of 89 million barrels per day in 2011 (International Energy Agency, http://omrpublic.iea.org/). Approximately, a half of this amount is transported by the sea (Gertler et al., 2010). As follows, worldwide marine coastal areas are exposed to the oil spills occurring as a result of accidents or illegal practices (Psarros et al., 2010). The release of thousands of tons of petroleum hydrocarbons (PHs) affects the marine environment and causes severe ecological and economical damage. For example, only the pollution resulting from the tanker washing or ballast water has been estimated to contribute about 2 million tons per year worldwide (Ferraro et al., 2007; Gertler et al., 2010). The recent spillage of 780,000 m^3^ of oil into the Gulf of Mexico proved again that human activities might cause a contamination without precedents. This accident presented a huge challenge to existing oil spill treatment methods, and current technologies were not able to cope with the size and nature of the Deepwater Horizon oil spill. Therefore, there is an urgent demand for development and optimization of bioremediation techniques that can play a central role in marine oil spill response contingency plans.

One of the most important issues in bioremediation is the application (or stimulation) of autochthonous hydrocarbon-degrading microbial populations. Some marine gammaproteobacteria have a high affinity toward PHs. Species such as *Alcanivorax borkumensis* (Yakimov et al., 1998), *Cycloclasticus pugetii* (Dyksterhouse et al., 1995), *Oleispira antarctica* (Yakimov et al., 2003), *Oleiphilus messinensis* (Golyshin et al., 2002), and *Thalassolituus oleivorans* (Yakimov et al., 2004) constitute a distinct group of obligate marine hydrocarbon-degrading bacteria (OMHCB). Following a sudden oil spill event, these organisms outcompete most of the naturally occurring oligotrophic marine microorganisms (Hara et al., 2003; Yakimov et al., 2007). Growing on PHs, aerobic OMHCB use oxygen not only as the terminal electron acceptor for respiratory energy conservation, but also as an indispensable reactant in the PHs activation mechanism. Thus, the stimulation of the OMHCBs degradation activity in the contaminated site can significantly improve the self-cleaning potential. Unfortunately, due to metabolic requirements of the OMHCBs, this type of bioremediation is restricted to either seawater column or superficial sediments. Due to a high biological oxygen demand and its slow diffusion into marine sediments, these compartments below the surface are typically highly reduced (Engelen and Cypionka, 2009). The realization that activated oxygen is used to overcome the chemical sluggishness of hydrocarbons has for some decades favored the view that hydrocarbons are not biodegradable under anoxic conditions (Widdel and Rabus, 2001). Although recently, a number of strictly anaerobic microorganisms have been shown to utilize PHs as growth substrates, this process is extremely slow compared to aerobic degradation and can not be considered as rapid bioremediation scenario.

The aim of this study was to monitor both immediate and long-term responses of indigenous microbial consortia to a simulated oil spill and during bioremediation treatment. Keeping in mind that the decontamination of PHs-polluted anoxic sediments is a very sluggish process, the stimulation of indigenous aerobic OMHCBs was performed by the *in situ* aeration of sediments within a modular slurry system (MSS) to avoid the contamination of the surrounding aquifer. The succession of microbial community and efficacy of petroleum biodegradation in both untreated (anoxic) external and aerated internal sediments was monitored during 3 months after contamination. Additionally, the toxicity of sediments was controlled by application of Microtox® and amphipods *Corophium orientale* eco-toxicological bioassays.

## Materials and methods

### Experimental mesocosm

**Figure 1:**
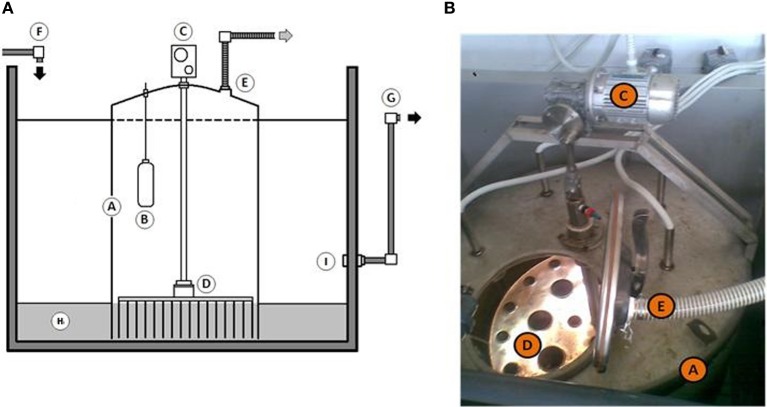
**(A,B)** Schematic representation **(A)** and detail **(B)** of “Modular Slurry System” used throughout this study. Abbreviation used: A, Modular Slurry System; B, temperature controller; C, external air pump; D, steel plate with needles to supply an oxygen into deep sediments; E, exhaust tube; F, seawater inlet; G, seawater outlet; H, contaminated sediments; I, overflow regulation system.

The experiment was carried out in rectangular tank of 3.75 m^3^ capacity (166 cm long, 150 cm deep, 150 cm wide). This reservoir was filled with ca. 2000 l of seawater taken directly from the harbor of Messina (38°11′42.58″N 15°34′25.19″E). Prior to use, the seawater was filtered through a 200 μm nylon mesh to remove large metazoans and detritus. Approximately 1000 kg of sandy sediments were collected at the same place and artificially contaminated with Bunker C furnace fuel oil (6500 ppm) to simulate the oil spill accident. Temperature inside the mesocosm was maintained about 20 ± 1°C for all experimental period. Mesocosm has continuously received seawater at the flow rate of 1 l min^−1^. The MSS used in mesocosm experiment is shown on Figure 1. The MSS was developed especially for *in situ* aeration (20 l min^−1^) of polluted sediments without their removal from contaminated side to avoid the re-contamination of adjacent aquifer. The reactor was inserted into the sediment. Sediment directly beneath the MSS were treated by the reactor, and those sediments outside of it were undisturbed and served as a control. All experimentations have been conducted for 3 months. To monitor the succession of microbial population and the efficiency of petroleum degradation, 1.5–2.0 kg of sediments (up to 10 kg in total) were sampled on fixed days (T_0_, T_1_, T_29_, and T_90_) at six different points inside and outside the MSS. Additionally, measurements of the biochemical oxygen demand (BOD_5_), reduction potential (E_h_), and eco-toxicological assays (Microtox® and *Corophium orientale* mortality test) were monitored. The E_h_ of sediment was measured by a Waterproof CyberScan PCD 650 multiparameter (Eutech Instruments) according to the manufacturer's instructions at aforementioned time intervals. BOD_5_ was measured using a BOD sensor (VELP Scientifica) after 5 days of incubation in accordance with the manufacturer's instructions.

### Analysis of petroleum hydrocarbons

Efficiency of petroleum degradation was estimated by the analysis of total extracted and resolved hydrocarbons (TERHC). At the fixed time points, TERCH were extracted from sediments following the 3550C EPA (Environmental Protection Agency) procedure. Briefly, 500 ml mixture of CH_2_Cl_2_: CH_3_COCH_3_ (1: 1, vol/vol) was added to 1000 g of pooled and dried either internal or external sediments, collected from six different sites (see above). Mixture was sonicated for 2 min in ultrasound bath (Branson 1200 Ultrasonic Cleaner, Branson USA). Samples were further shaken at 150 rpm for 30 min, centrifuged for 10 min at 5000× g and supernatant was passed through a ceramic column filled with anhydrous Na_2_SO_4_ (Sigma-Aldrich, Milan). Same treatment of pooled and dried sediments was repeated with 500 ml of CH_2_Cl_2_ and the obtained extracts were combined and volatilized to the dryness. Residues were re-suspended in CH_2_Cl_2_ prior the gas chromatography (GC) analysis (Rocchetti et al., 2011). All measures were performed using a Master GC DANI Instruments (Development ANalytical Instruments), equipped with SSL injector and FID detector. Sample (1 μl) was injected in splitless mode at 330°C. The analytical column was a Restek Rxi-5 Sil MS with Integra-Guard, 30 m × 0.25 mm (ID × 0.25 μm film thickness). Helium carrier gas was maintained at a constant flow of 1.5 ml min^−1^. TERCH were calculated using the mean response factors of *n*-alkanes, i.e., individual *n*-alkane concentrations from *n*-C_15_ to *n*-C_40_, pristane and phytane were calculated for each sample. To estimate the biodegradation of aliphatic fraction, the evaluation indices *n*-C_17_/pristane and *n*-C_18_/phytane were selected for this study. The amount of analyzed TERCH was expressed as ppm (part per million) or mg kg^−1^.

### Ecotoxicological assays

The Microtox® luminescence assay was performed on sediment pore water. Sub-samples of sediment were centrifuged (5000× g for 45 min, 4°C) and filtered (0.45 μm nitrate cellulose membrane) to remove the fine suspended particles and maintained at 4°C until used in assays. Microtox® toxicity tests were conducted according to the standard procedures EN12457 with the following modifications. As far as the Solid Phase Test deals with fine particles that affected the bioluminescence of bacteria (Bulich et al., 1992; Benton et al., 1995; Ringwood et al., 1997), each experimental sediment sample was compared with a reference sediment sample with the same granulometry, collected from a pristine site. Toxicities were reported as effective concentration of toxicant resulting in a 50% decrease in bioluminescence (EC_50_). EC_50_ with 95% of confidence intervals were calculated following the procedures outlined in the Microtox® System Operating Manual (Microtox, 1982). Amphipods *Corophium orientale* were delivered from CIBM (Livorno, Italy). The animals were used following the procedure reported by Onorati et al. (1999). Briefly, the juveniles and young adults, which passed through 1000 μm and retained by 710 μm mesh sieve, were selected for ecotoxicology experiments. The test was carried out inside 2.5-l glass flasks containing approximately 2 cm layer of sediments and filled with 1000 ml of filtered seawater. The seawater was aerated and kept at a constant temperature (16 ± 2°C). Flasks were illuminated during 12 h daily by a lamp system consisted of six tubes (36W, 120 cm). One hundred amphipods were randomly selected and introduced into each flask. No food was added to the test and control chambers. At the end of exposition (10 days), amount of survived organisms were counted. Missing amphipods were assumed as dead animals. The sensitivity of the populations was estimated as a fraction of dead organisms to the initial amount of added amphipods. All the experiments were replicated twice.

### Total RNA extraction, reverse transcription-PCR (RT-PCR) and 16S rRNA clone libraries

Total RNA was extracted from sediment sample (5 g) using the FastRNA® Pro soil direct Kit (MP Biomedicals™) as previously described (Roussel et al., 2009). Extracted RNA from three different samples was pooled and further converted to cDNA using First-Strand cDNA Synthesis SuperScript™ II Reverse Transcriptase (Life Technologies). RT reaction mixtures (20 μl) contained 1 μl the random hexamer primer mix (Bioline), 30 ng of RNA, 1 μl 10 mM dNTPs (Gibco, Invitrogen Co., Carlsbad, CA) and sterile distilled water (14 μl). After heating to 65°C for 5 min, and incubate on ice for 1 min, the mixture was supplemented with 4 μl of 5 × First-Strand Buffer, 1 μl of DTT and 1 μl of SuperScript™. The mixture was shaken and incubated at 50°C for 30 min. Finally, SuperScript™ enzyme was inactivated by heating at 70°C for 15 min. 16S rRNA genes were amplified from total cDNA using the universal primes (530F [5′-GTGCCAGCMGCCGCGG-3′] and 1492R [5′-TACGGYTACCTTGTTACGACT-3′]) (Lane, 1991). The PCR was performed in 50 μ l mixture (total volume) containing 1× solution Q (Qiagen), 1× Qiagen reaction buffer, 1 μM of each forward and reverse primer, 10 μM dNTPs (Gibco, Invitrogen Co.), 2.0 ml (50–100 ng) of template and 2.0 U of Qiagen Taq Polymerase (Qiagen). The reaction (3 min hot-start at 95°C; 1 min at 94°C, 1 min at 50°C, 2 min at 72°C, 30 cycles; final extension 10 min at 72°C) was performed with GeneAmp 5700 (PE Applied Biosystems). The quality of amplification products was checked by agarose electrophoresis and purified using Qiaquick Gel Extraction kit (Qiagen). Purified 16S crDNA amplicons were further cloned into the pGEM T-easy Vector II (Promega), transformed into *E. coli* DH10β cells and subsequently amplified with primers, specific for the pGEM T-easy vector (M13F (5′-TGTAAAACGACGGCCAGT-3′) and M13R (5′-TCACACAGGAAACAGCTATGAC-3′). Positive products were purified and sequenced by Macrogen (Amsterdam, The Netherlands). Sequences were checked for possible chimeric origin using Pintail 1.1 software (Ashelford et al., 2005). For the 16S rRNA gene sequences, initial alignment of amplified sequences and close relatives identified with BLAST (Altschul et al., 1997) were performed using the SILVA alignment tool (Pruesse et al., 2007) and manually aligned with ARB (Ludwig et al., 2004). After alignment, the neighbor-joining algorithm of ARB package was used to generate the phylogenetic trees based on distance analysis for 16S rRNA genes. The robustness of inferred topologies was verified by bootstrap re-sampling analysis, using the same distance model (1000 replicates).

### Enumeration of cells by card-fish

The catalyzed reporter deposition fluorescence *in situ* hybridization (CARD-FISH) was performed using the protocol of Pernthaler et al. (2002) with some modifications. Briefly, 5 g of pooled sediments samples were fixed with formaldehyde (2% v/v final concentrations) and left for 12 h at 4°C. Fixed sediment samples were further incubated for at least 15 min with Tween 80 (final concentration, 1 mg l^−1^) and than sonicated during 20 min in an Ultrasonic cleaner (Branson 1200, Milan) for the bacterial dispersion (Kuwae and Hosokawa, 1999). Supernatant samples were then filtered onto polycarbonate membrane filters (type GTTP; pore size, 0.2 μm; diameter, 2.5 cm; Sartorius, Göttingen, Germany). Filters for CARD-FISH counts were embedded in low-melting point agarose (0.1% wt/vol, Sigma-Aldrich, Milan), dried at 37°C for 20 min, and dehydrated with 95% ethanol. Embedded cells were permeabilized by 1 h of exposition with solution A (10 mg ml^−1^ of lysozyme (Sigma-Aldrich, Milan); 0.5 M EDTA, 0.1 M Tris-HCl [pH 8.0]) followed by the 30 min-long incubation with achromopeptidase (60 U ml^−1^; 0.01 M NaCl, 0.01 M Tris-HCl [pH 8.0]) at 37°C. Filters were cut in sections and hybridized with 5′-horseradish peroxydase (HRP)-labeled oligonucleotide probes as described by Pernthaler et al. (2002). Probes used in this work are listed in Table 1. After the hybridization and amplification steps, slides were examined using a epifluorescence with Axioplan 2 Imaging (Zeiss; Carl Zeiss Inc., Thornwood, NY) microscope. All results were expressed as number of cells per gram of sediment.

**Table 1:** **CARD-FISH specific probes used in the present study**.

**Probe**	**Sequence (5′ to 3′) of probe**	**FA (%)**	**Source**
Eub338	GCTGCCTCCCGTAGGAGT	35	Amann et al., 1990
*Marinobacter* sp.	ATGCTTAGGAATCTGCCCAGTAGTG	20	Karner and Fuhrman, 1997
*Cycloclasticus* sp.	GGAAACCCGCCCAACAGT	20	Karner and Fuhrman, 1997
*Alcanivorax* sp.	CGACGCGAGCTCATCCATCA	20	Karner and Fuhrman, 1997

FA: percentage of formamide in hybridization buffer.

### Determination of cell numbers by quantitative PCR (qPCR)

qPCR method was employed to determine the relative cell densities of *Alcanivorax, Marinobacter*, and *Cycloclasticus* in the sediment samples. Primers used through this study are listed in the Table 2. Primers were based on the sequences of *Alcanivorax* alkane hydroxylase gene (*alkB2*), *Marinobacter* alkane hydroxylase gene (*alkB*), and *Cycloclasticus* aromatic ring-hydroxylating dioxygenase (*phnA*) gene. Primers for *alKB2* and *phnA* genes were previously designed and validated for qPCR elsewhere (McKew et al., 2007; Gray et al., 2011). The primers specific for the *alkB* gene of *Marinobacter* were designed through this study using Primer Express software Primer Express software, version 2.0 (Applied Biosystems, Foster City, Calif.) with reference to the *Marinobacter hydrocarbonoclasticus* (FO203363).

**Table 2:** **qPCR primers used in the present study**.

**Gene**	**Forward Reverse**	**Organism**	**Amplification efficiency (E)**	**Source**
*alkB2*	CGCCGTGTGAATGACAAGGGG	*Alcanivorax*	99.6%	McKew et al., 2007
	CGACGCTTGGCGTAAGCATG			
*alkB*	TCCTTTGGTATGGCGCAGTT	*Marinobacter*	97.3%	This study
	ACGATCCTGTTCAAGCCGAG			
*phnA*	CGTTGTGCGCATAAAGGTGCGG	*Cycloclasticus*	96.2%	McKew et al., 2007
	CTTGCCCTTTCATACCCCGCC			

Total DNA was extracted from 2.0 g of sediment samples collected at selected time scales from three different points inside the MSS by using a Bio101 FastDNA SPIN kit as described by the manufacturer (Bio101, Inc., Vista, Calif.). Extracted DNA was dissolved in 50 μ l of TE buffer (10 mM Tris-HCl, 1 mM EDTA [pH 7.5]) and quantified using a NanoDrop ND-1000 spectrophotometer (Celbio). The quality of the extracted DNA was analyzed by electrophoresis on a 1.0% agarose gel. The qPCR was performed with absolute quantification method in an ABI Prism 7300 Real-time PCR System (Applied Biosystems) in a total volume of 25 μ l, consisting 12.5 μ l of SYBR green master mix, 200 nM of each primers, and 50 ng of template DNA. The volume of each reaction was adjusted to 25 μ l by adding DNase-free water. The cycling parameters for the qPCR amplification were as follows: an initial denaturation step at 95°C for 10 min, followed by 45 cycles of denaturation at 95°C for 15 s and annealing/elongation at 60°C for 60 s. A dissociation step was added to check for primer-dimer formation. A tenfold serial dilution series of genomic DNA ranging from 10 to 10^8^ copies per reaction was used in triplicate to create the standard curve for quantification. Serial dilutions were prepared once for each target and used for real-time quantification. From the slope of each curve, PCR amplification efficiency was calculated as it described elsewhere (Rasmussen, 2001). Obtained slope values, (−3.17 for *alkB2*, −3.29 for *alkB*, and −3.37 for *phnA*) fell within the optimal range corresponding to an efficiency of 99.6, 97.3, and 96.2%, respectively. Amplicon numbers were quantified against the standard curve using automatic analysis settings for the Ct values and baseline settings. Detected target genes were converted to cell density in sediments (cells gram^−1^) assuming that all three genes present as a single copy per genome.

### Statistical analysis

For statistical analyses, clones from each 16S crDNA library were separately considered to define phylotypes, or operational taxonomic units (OTUs) at cutoff of either 95 or 97% of sequence identity (Kemp and Aller, 2004). Clustering of sequences was performed using Dotur program (Schloss and Handelsman, 2005). Various parameters for each clone library, such as diversity index, rarefaction analysis, taxa, total clones, singletons, Shannon dominance, equitability, Simpson and chao2 were calculated by PAST version 2.17c (http://folk.uio.no/ohammer/past; Hammer et al., 2001). Coverage values given as C = 1 − (n_1_/N), where n_1_ is the number of clones which occurred only once in the library of N clones (Good, 1953), were calculated to determine how efficient clone libraries described the complexity of original bacterial community. The Primer 6 ecological software package developed by the Plymouth Marine Laboratory (Clarke and Gorley, 2006) was employed to perform the Hierarchical Cluster Analysis (HCA) (Clarke, 1993). We applied HCA on microbial biodiversity and Bray-Curtis similarity on relative abundance matrix of the OTUs detected at different sampling time. Significant difference of the microbial assemblages derived from both treated and control samples at the different sampling times was detected via the *P*-test significance and principal coordinates analysis (PCoA) using UniFrac program (http://bmf.colorado.edu/unifrac/index.psp, last access: 24 July 2008) (Lozupone et al., 2007). Differences in cell number per gram of sediment detected by DAPI, CARD, and qPCR between different samples was examined by analysis of variance (ANOVA) on ranks (Holm_Sindak method). Relative importance of each treatment group was investigated by Multiple Comparisons vs. Control Group comparison test. Calculations were carried out using SigmaStat software for Windows, ver. 3.1 (Copyright 1992–1995; Jandel Corporation). Differences were considered significant at *P* < 0.05.

### Nucleotide sequence accession numbers

To avoid the submission of identical sequences obtained among 411 analyzed clones, only 98 distinguishing 16S rRNA gene sequences have been deposited in the DDBJ/EMBL/GenBank databases under accession numbers KF896304-KF896401.

## Results

### Geochemical properties of the sediments

**Figure 2:**
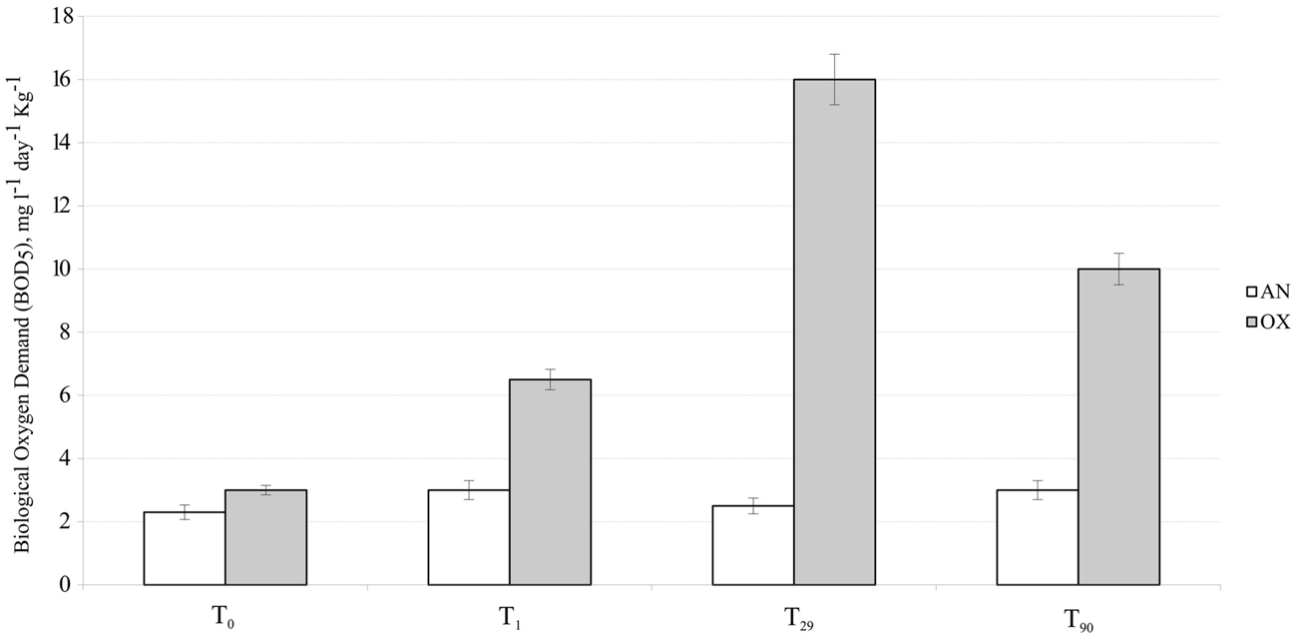
**Dynamic of oxygen consumption (BOD values) measured in MSS external (untreated) superficial sediments (white bars) and internal sediments (gray bars)**. Error bar indicates the standard deviation of triplicate measurements.

**Figure 3:**
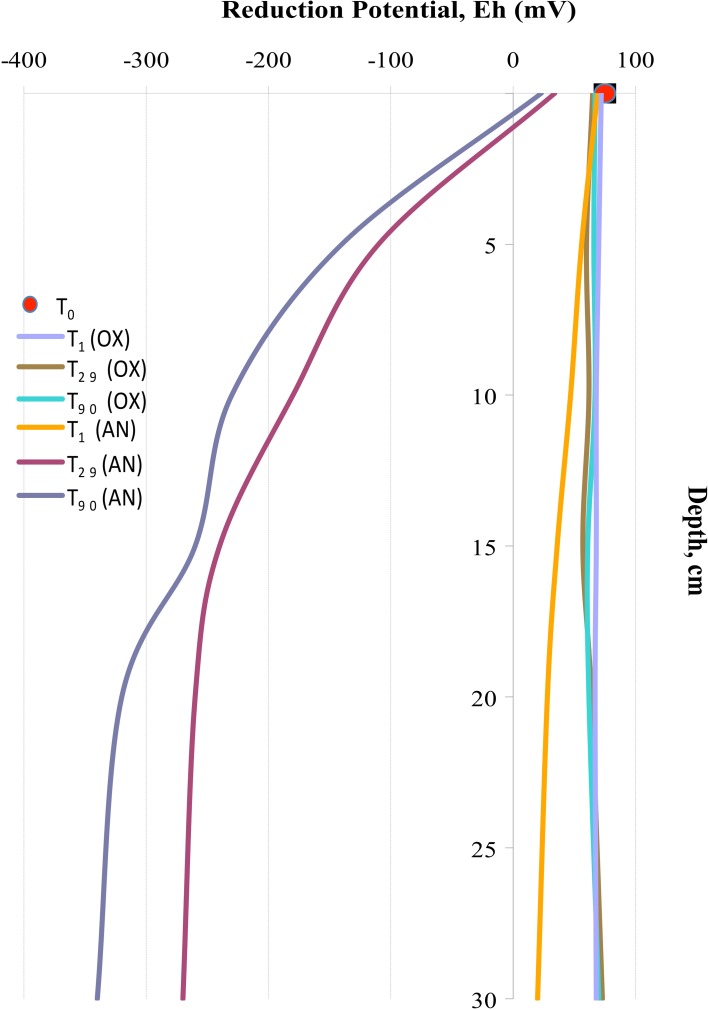
**Reduction potential (E_h_) measured in external anaerobic sediments (AN) and in internal aerated sediments (OX) during 3 months of experimentation**.

Oxygen consumption in the external superficial sediments (0–5 cm) was monitored during all 3 months of experimentation. These values were compared with BOD in the internal MSS sediments taken at the beginning (T_0_), after 1 day (T_1_), 1 month (T_29_), and after 3 months (T_90_) of experimentation (Figure 2). Sediments outside of the MSS exhibited constant BOD rates of approximately 2.5 mg O_2_ day^−1^ kg^−1^ during all period of experimentation, whereas the aerated sediments inside of the MSS demonstrated a progressive increment of oxygen consumption. Maximum of oxygen demand (16.0 mg O_2_ day^−1^ kg^−1^) was obtained at T_29_ and afterwards the BOD values trended to diminish, reaching 10.0 mg O_2_ day^−1^ kg^−1^ at the end of experiment. Outside the MSS, the amendment of the Bunker C furnace fuel oil turned initially oxygenated (T_0_, E_h_ = 77 ± 4 mV) sediments into highly reduced ones (Figure 3). The external sediments below 5 cm became oxygen-depleted already within 1 day after spiking, obviously due to the active respiration of aerobic heterotrophic microorganisms. The reduction potential of external sediments decreased continuously during the experiment and after 3 months reached the E_h_ values of −345 mV in the deepest layers. In contrary, inside the MSS the sediments remained aerobic during all period of bioremediation effort.

### Hydrocarbon analysis

Before the addition of 6500 ppm of Bunker C fuel oil into the mesocosm, the total hydrocarbon concentration in the original Messina harbor sediments was estimated at the level of 120 ppm. The PHs fingerprint analysis showed a clear dominance of alkyl-aromatic derivatives (97%) over aliphatic hydrocarbons (3%) (data not shown). Once Bunker C fuel oil was added to the sediments, the TERHC fraction mass balance was shifted toward the dominance of aliphatic and naphthenic hydrocarbons (70%) over aromatics (30%). The degree of the Bunker C fuel oil degradation in both, aerated MSS and in the untreated anoxic sediments was examined at the end of experiment (T_90_). The concentration of PHs, especially aliphatic hydrocarbons was normalized using the pristane/phytane ratio, and the values obtained in triplicate subsamples were averaged. In the aerated internal MSS sediments, the total degradation of Bunker C fuel oil TERHC fraction was 97.7 ± 0.9%. In contrast, external anoxic sediments contained more than 81.8 ± 1.2% of initially added Bunker C fuel oil. Additionally, we performed the gravimetrical analysis of total extracted hydrophobic fraction (TEHF) in the sediments. The TEHF values inside the MSS accounted for 780 ± 80 mg kg^−1^ dry sediment weight, whereas in concordance with extremely slow biodegradation rates under anaerobic conditions, the external sediments contained more than 5400 ± 120 mg kg^−1^ of hydrophobic material. As shown in subsequent section, TEH fraction seems be primarily responsible for the observed toxicity of Bunker C fuel oil.

### Ecotoxicological analysis

**Figure 4:**
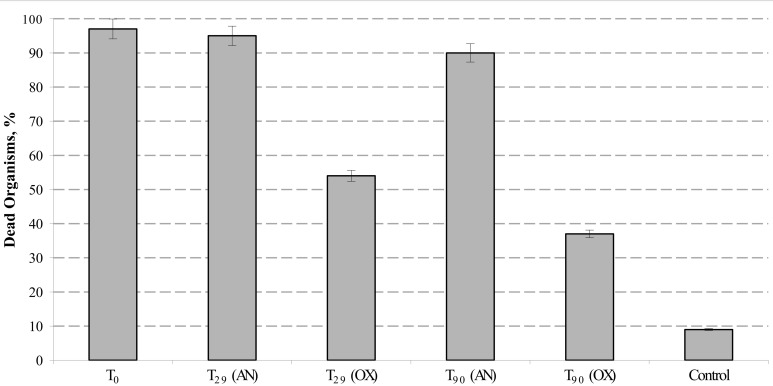
**Mortality of *Corophium orientale* organisms in polluted (AN), treated (OX), and control (native) sediments**. Error bar indicates the standard deviation of duplicate measurements.

According to standard guidelines of Italian Institute for Environmental Protection and Research (ISPRA, 2013), eco-toxicological analysis of hydrocarbon-contaminated sediments were carried out using the Microtox® luminescence and amphipod *Corophium orientale* bioassays. Eventual decrease in Microtox® bioluminescence was measured on sediment pore water, whereas the rate of amphipods mortality was tested by direct exposition of *C. orientale* with the petroleum-contaminated sediments during 10 days. Following the EN12457 protocol, we combined the sediment pore water with sterile seawater in both 1:2 and 1:10 ratios (vol/vol) and no significant level of bioluminescence decay has been observed. As it reported in EN12457 protocol, Microtox® bioluminescent assay tested on sediment pore water typically exhibits an underestimated sensitivity against highly hydrophobic contaminants, such as PHs. This is mainly due to both extremely low solubility of these compounds in water and an almost irreversible adsorption to a sedimentary matrix. Corresponding to the standardized protocol described by Onorati et al. (1999), the toxicological analysis of the sediments was performed with amphipods *C. orientale*. Addition of Bunker C fuel oil to the sediments (T_0_) caused the mortality in almost all organisms (98 ± 2%). The petroleum-contaminated external sediments remained highly toxic during all 3 months of experimentation with mortality indices exceeding 90% (Figure 4). At the same time, the toxicity of polluted internal MSS sediments dropped almost twice after 1 month of aeration (T_29_) and continued to decrease till the end of bioremediation treatment, approaching the vitality of 62% of amphipods exposed to T_90_ sediments. This indicated that the internal MSS sediment at T_90_ was significantly less toxic than their external counterpart.

### Diversity and succession of bacterial communities during bioremediation and natural sediment ageing

**Figure 5:**
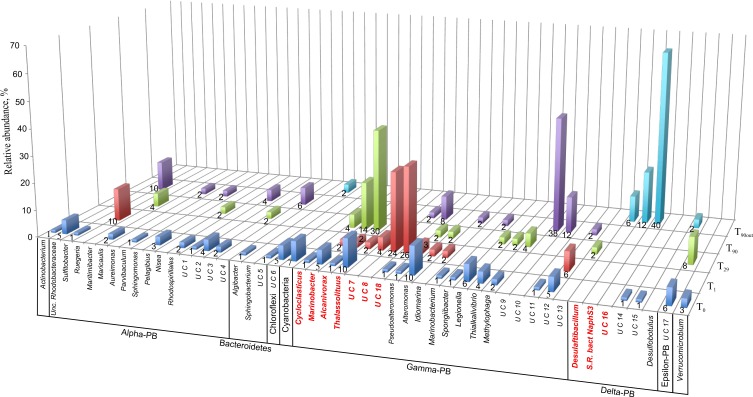
**Dendrogram representation of taxonomic analysis of 16S crDNA clones retrieved from five libraries**. The numbers at the base of columns represent the percentage of clones in corresponding libraries. Abbreviations used: PB, *Proteobacteria* divisions; UC, Unaffiliated cluster. Bacterial groups involved in, or associated to, hydrocarbon degradation are marked in bold and red.

**Figure 6:**
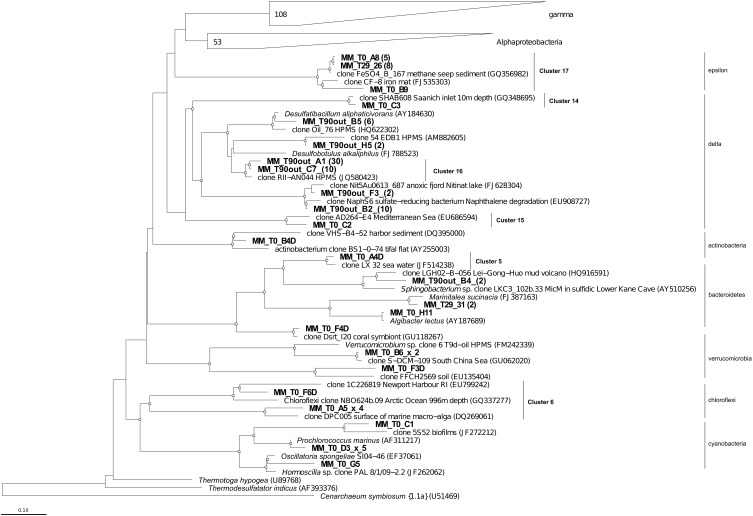
**Phylogenetic affiliation of the *Eubacteria* clones retrieved in Messina harbor native sediments and during mesocosm experiment**. Neighbor-joining analysis using 1000 bootstrap replicates was used to infer tree topology. The scale bar represents 10% of sequence divergence. Bootstrap values ≥50 are shown as open circles. *Cenarcheum symbiosum* was used as the outgroup. The scale bar represents the expected number of changes pet nucleotide position. Sequences from this study are indicated in bold.

To monitor the succession of metabolically active microbial communities during the bioremediation treatment, five 16S rRNA transcript libraries were established. 450 clones from these libraries were randomly selected and analyzed. Among those, 411 sequences were included into phygenetic analysis (T_0_, 96 clones; T_1_, 83 clones; T_29_, 80 clones; T_90_, 90 clones and T_90OUT_, 62 clones). The majority of native T_0_ clones were affiliated with the *Gammaproteobacteria* (51%), followed by the *Alphaproteobacteria* (22%). Other proteobacteria, belonging to microaerophilic and anaerobic *Epsilon*- and *Deltaproteobacteria* were also present, although in significantly lower numbers (6 and 2% of all clones analyzed, respectively). Remaining fraction of T_0_ microbial community consisted of the members of *Cyanobacteria* (7%), *Chloroflexi* (5%), *Verrucomicrobia* (3%), and *Bacteroidetes* (2%) (Figure 5). At the level of the Class, the derived from members of *Gammaproteobacteria* were predominant in all analyzed MSS internal sediments, with percentage ranging from 73 to 88%. There was a 50%-reduction of *Alphaproteobacteria* observed during first month of sediment treatment (decrease from 22 to 11%). Further on, their numbers returned to the initial (T_0_) values by the end of experiment. No *Deltaproteobacteria*–related organisms were detected in the internal MSS sediments throughout the experiment. A completely different scenario was observed regarding the succession of microbial community thriving in the external anoxic sediments. As we mentioned above, after loading the Bunker C fuel oil, the sediments became highly reduced within a short period of time and were inhabited mainly by the members of *Deltaproteobacteria* (96.8%). The analysis of 16S rRNA transcripts from T_90OUT_ clone library revealed a notable prevalence of hydrocarbon-degrading or hydrocarbon contamination-associated *Deltaproteobacteria* (90.3%). In particular, almost two-thirds of all clones (40 out of 62 clones analyzed) were closely related to the uncultured bacterium RII-AN044 found in anoxic polluted sediments after the Prestige oil spill (Acosta González et al., 2013). More than 16% of clones revealed >98% of similarity to the deltaproteobacterium NaphS6, capable of naphthalene and 2-methylnaphthalene degradation (Wilkes et al., 2008). Almost 10% of T_90OUT_ clones were closely related to the *n*-alkanes- and *n*-alkenes-degrading strain *Desulfatibacillum aliphaticivorans* (Cravo-Laureau et al., 2004) (Figure 6).

**Figure 7:**
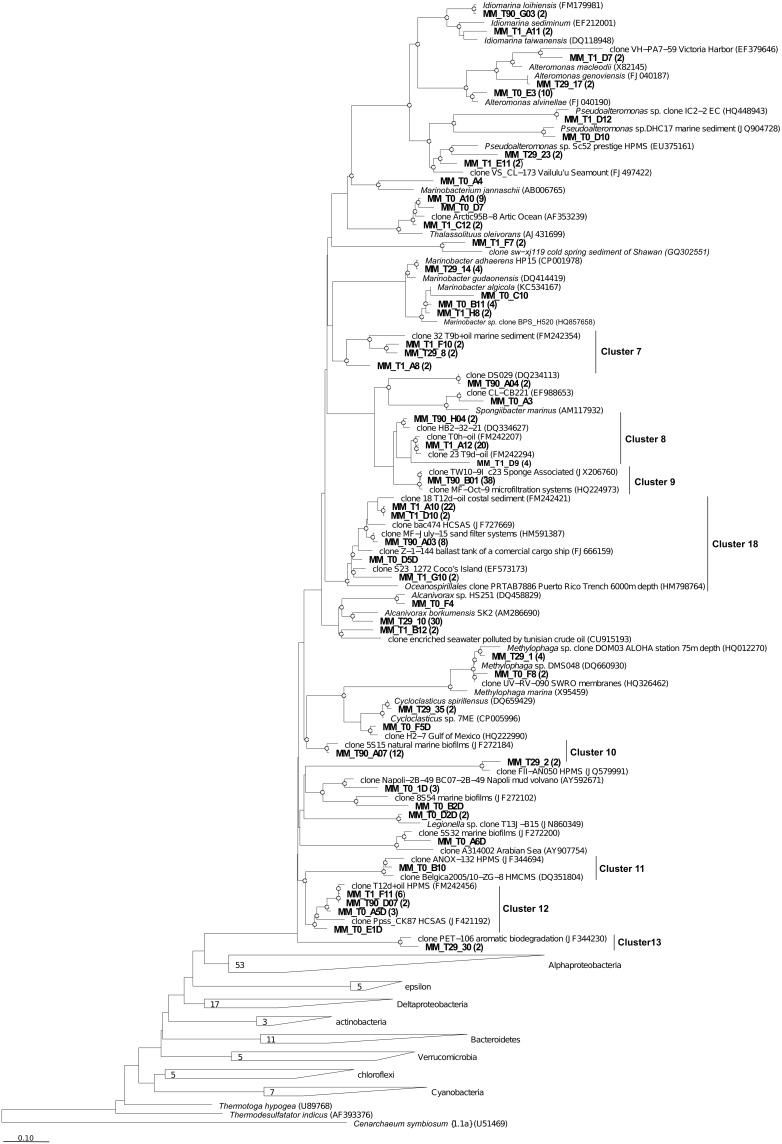
**Phylogenetic affiliation of the *Gammaproteobacteria* clones retrieved in Messina harbor sediments and in mesocosm experimentation.** Neighbor-joining analysis using 1000 bootstrap replicates was used to infer tree topology. The scale bar represents 10% of sequence divergence Bootstrap values ≥50 are shown as open circles. *Cenarcheum symbiosum* was used as the out-group. Sequences obtained in this study are indicated in bold.

**Figure 8:**
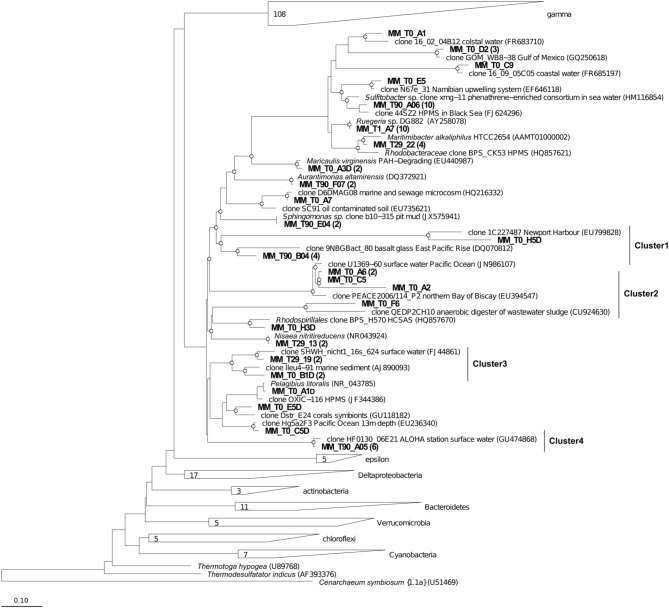
**Phylogenetic affiliation of the *Alphaproteobacteria* clones retrieved in Messina harbor sediments and in mesocosm experimentation.** Neighbor-joining analysis using 1000 bootstrap replicates was used to infer tree topology. The scale bar represents 10% of sequence divergence. Bootstrap values ≥50 are shown as open circles. *Cenarcheum symbiosum* was used as the outgroup. Sequences obtained in this study are indicated in bold.

In accordance with the type of the contaminant load, the majority of analyzed clones had highest Blastn homologies with sequences related to PH-degrading or petroleum contamination-associated organisms belonging to the *Gamma*-, *Alpha*- and *Deltaproteobacteria* (Figures 6–8). At the genus and species level, more than 10% of initial microbial population was attributed to obligate marine hydrocarbonoclastic bacteria *Thalassolituus oleivorans* (Yakimov et al., 2004). This organism seemed to be sensitive to Bunker C fuel oil, since its abundance decreased to 2% after 1 day of oil exposition and disappeared afterwards. Three other OMHCB belonging to genera *Alcanivorax*, *Cycloclasticus*, and *Marinobacter* demonstrated similar dynamics, i.e., being in relative minority in the beginning of the experiment, they became predominant in T_29_ microbial community and disappeared in the T_90_ library (Figure 5). Noteworthy, the addition of Bunker C fuel oil to sediments drastically changed the structure of T_1_ microbial community and the proportion of aforementioned *Gammaproteobacteria*-related OMHCB decreased threefold compared with the initial population. In contrast, previously undetected organisms related to dinoflagellate-associated *Rugeria* sp. and to three deep-branching clusters of *Gammaproteobacteria* accounted for 77% of all analyzed T_1_ clones. Having only 16S rRNA gene sequences at our disposal, we cannot state that these uncultured organisms were involved in biodegradation activity, but they definitely possessed a remarkable resilience to the toxicity of Bunker C fuel oil (Païssé et al., 2008, 2010).

Additionally to 16S rRNA-based analysis, the diversity and succession of both, total bacterial population and hydrocarbon-degrading bacteria, was assessed through the combined application of CARD-FISH and qPCR. Using the *Eubacteria*-specific probe Eub338, the concentration of CARD-positive cells at the beginning of experiment was estimated at 2.98 ± 0.17 × 10^6^ cells gram^−1^ (Table 3). Their numbers decreased by 20% within 1 day after the oil spill simulation (*P* < 0.001, *n* = 10) and reached initial values at the end of experiment (2.79 ± 0.12 × 10^6^ cells gram^−1^). Noteworthy, a tenfold increase in the number of CARD-positive cells (2.95 ± 0.11 × 10^7^ cells gram^−1^) was detected after 29 days of oil spill, which fully corresponded to the observed dynamics of the BOD and clone libraries' values (Figure 2). Before the oil spill simulation (T_0_), the fraction of *Alcanivorax*-related cells, detected with the CARD-FISH genus-specific probe, accounted for 7.7% of all Eub338-positive cells. After the addition of Bunker C fuel oil, their abundance increased within 1 month up to 27.5%. According to the analysis of 16S crDNA clone libraries, *Alcanivorax* became extinct in microbial community thriving in the MSS internal sediments at the end of experiment. Dynamics of the *Marinobacter*-related bacteria was comparable with that of *Alcanivorax* population, with the only exception that the *Marinobacter* cells decreased their abundance by 44% at T_1_, likely due to the higher sensitivity to the load of fuel oil. Compared to an initial density, their population increased twofold at T_29_, from 4.5 ± 0.2 to 9.0 ± 0.1 × 10^5^ cells g^−1^. Although due to the overwhelming growth of *Alcanivorax*, the relative abundance of *Marinobacter* during bioremediation experiment has never exceeded its initial values (15.2% of all Eub338-stained cells at T_0_) and at T_29_ (corresponding to the maximum of cell density in MSS) accounted for only 3% of total microbial community. Bacteria stained with *Cycloclasticus*-specific CARD-FISH probe, initially present in mesocosm sediments at concentration of 1.14 ± 0.15 × 10^5^ cells gram^−1^, were not detected at T_1_, whereas their concentration increased four times after 1 month of the oil spill simulation. Similarly to the dynamics of *Alcanivorax*, neither *Marinobacter*, nor *Cycloclasticus* were present in the MSS microbial community at the end of experiment.

**Table 3:** **CARD-FISH and qPCR cell number quantification in the MSS internal sediment during the bioremediation treatment**.

**Method**	**Cell numbers, 10**^5^** × g sediments**^−1^** ± *SD***				
	**Probe/primers**	**T**_0_****	**T**_1_****	**T**_29_****	**T**_90_****
CARD-FISH	*Eubacteria*	29.8 ± 1.7	23.6 ± 1.5	295.0 ± 11.0	27.9 ± 1.2
	*Alcanivorax* sp.	2.3 ± 0.1	3.1 ± 0.2	81.0 ± 1.3	ND
	*Marinobacter* sp.	4.5 ± 0.2	2.6 ± 0.1	9.0 ± 0.1	ND
	*Cycloclasticus* sp.	1.2 ± 0.2	ND	4.9 ± 0.1	ND
qPCR^*^	*alkB2*	5.17 ± 0.17	4.73 ± 0.18	94.60 ± 3.80	ND
	*alkB*	7.90 ± 0.23	3.50 ± 0.20	15.30 ± 2.00	ND
	*phnA*	2.10 ± 0.16	3.78 ± 0.12 × 10^−3^	7.13 ± 0.28	ND

* These values mean the average number of cells detected in triplicate from three individual subsamples of sediments collected in different parts of MSS.

Succession of hydrocarbon-degrading bacteria during the sediment bioremediation was additionally quantified by qPCR. Based on the a priori higher sensitivity of qPCR approach, obtained numbers were slightly higher than those from taxon-specific CARD-FISH data. Nevertheless, obtained results remarkably corroborated with CARD-FISH counts and the general trend in *Alcanivorax*, *Marinobacter*, and *Cycloclasticus* dynamics was identical (Table 3). None of these hydrocarbon-degrading bacteria were detected by qPCR at the end of experiment.

### Statistical analysis of bacterial diversity

**Figure 9:**
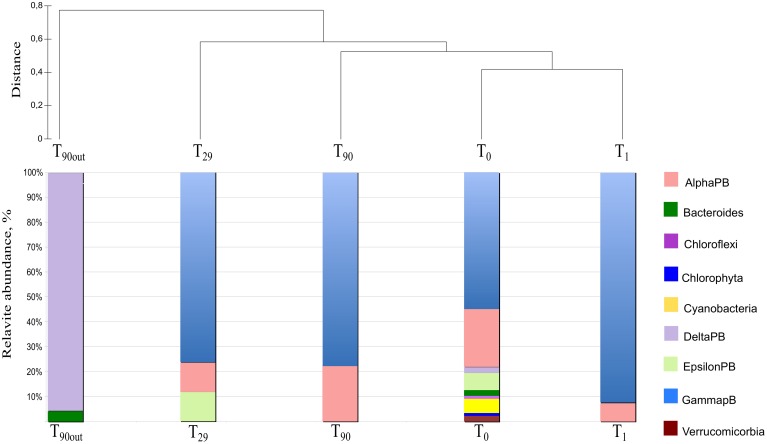
**Dendrogram of microbial biodiveristy and similarity analysis of the 16S rRNA transcripts detected at different sampling time and treatment.** The UPGMA cluster analysis was obtained by using group average clustering from Euclidean distance on relative abundance matrix of OTUs detected in the analyzed libraries.

As it shown in Table 4, the diversity index and coverage values have been calculated for each 16S rRNA transcript library. Based on sequence similarity threshold for OTU definition ≥97% (Rosselló-Mora and Amann, 2001), a total of 93 different OTUs were discerned. The rarefaction analysis did not demonstrate the saturation in any library (data not shown), however, the coverage values varying between 0.64 (T_0_ library) and 0.92 (T_9 0OUT_ library) indicated that a satisfactory overview on bacterial community was obtained. As reflected by the high values of Simpson, Shannon and the number of missing species provided by Chao-2, the highest bacterial diversity was observed in original sediments (T_0_). Bacterial biodiversity was drastically affected by Bunker C fuel oil addition, decreasing the Simpson, Shannon and Equitability values, due to strong selection for both petroleum contamination-resilient and hydrocarbon-degrading bacteria (Harayama et al., 1999; Kasai et al., 2001, 2002; Syutsubo et al., 2001). Dominance index reached the highest value at T_29_ and T_90_. After 1 month of aeration, the microbial population of the internal MSS sediments was dominated by OMHCBs, whereas at the end of experiment, the dominant Cluster 9 of the *Gammaproteobacteria* accounted for 42% of all T_90_ clones. This cluster consists of organisms recovered from pristine seawater and marine sediments. As mentioned above, the microbial community of anaerobic external sediments T_90OUT_ was totally different compared with aerated internal sediments. Both fuel oil contamination and rapid development of anaerobiosis have selected a very specialized and poorly diverse microbial population (5 OTUs; Dominance 0.44; Coverage 91%). These data were confirmed by the HCA and UniFrac PCoA analyses. At the end of the experiment, the microbial communities thriving in the MSS external (T_90OUT_) and the internal (T_90_) sediments were significantly different from each other (P1 = 50.85% and P2 = 31.39%). Noteworthy, both applied statistical analyses confirmed that comparing with OMHCB-enriched T_29_ population, the T_90_ microbial community was more similar to those recovered from early stages of the experiment (T_0_ and T_1_ libraries) (Figure 9). This finding indirectly hints at the process of self-recovery of petroleum-contaminated sediments, which was confirmed by GC analysis of the contaminated sediments in and outside the MSS.

**Table 4:** **Diversity indices calculated for the five clone libraries created at different time of aeration and sediment ageing**.

**Diversity index**	**T**_0_****	**T**_1_****	**T**_29_****	**T**_90_****	**T**_90OUT_****
Taxa/(OTUs)_S	46	16	14	12	5
Number of clones	96	83	80	90	62
Dominance_D	0.04	0.15	0.22	0.24	0.44
Simpson_1-D	0.96	0.85	0.78	0.76	0.55
Shannon_H	3.5	2.31	2.08	1.89	1.09
Equitability_J	0.91	0.84	0.78	0.76	0.68
Chao-2	104.1	16	14	12	5
Singletons	31	1	0	0	0
Doubletones	7	10	8	6	2
Coverage	0.64	0.74	0.77	0.87	0.92

## Discussion

The influence of the massive Bunker C fuel oil load upon the microbial population of coastal sediments collected in Messina harbor and their recovery was investigated during 3 months in the mesocosms experiment. These sediments chronically polluted with alkylnaphthalenes were chosen since the autochthonous microbial community is likely adapted to the steady presence of PHs and consequently may exhibit a higher biodegradation ability than those from pristine environments (Païssé et al., 2008, 2010). Accordingly, the fraction of the OMHCB genera *Alcanivorax Cycloclasticus Marinobacter* and *Thalassolituus*, usually imperceptible in pristine marine environments (Yakimov et al., 2007), accounted for a fifth part of the native microbial community of Messina harbor sediments. To simulate accidental oil spill we spiked 1000 kg sandy sediments with Bunker C fuel oil (6500 mg kg^−1^). This type of heavy fuel oil is frequently used by the cargo and tanker ships and is generally more complex in composition and impurities than distilled fuels. Bunker C fuel oil includes about 25% polyaromatic, 15% aliphatic, 45% naphthenic and 15% non-hydrocarbon compounds (Clark et al., 1990) As it generally observed, after the oil spill reaches a shoreline, the oil burial is the main mechanism of the pollution dispersal. This results in intense oiling of subsurface sediments and consequent reduction in oxygen concentration due to initial activation of aerobic hydrocarbon-degrading microorganisms (Albaigés et al., 2006; Acosta González et al., 2013). Once O_2_ concentration drops to zero, the degradation rates of both aliphatic and aromatic PHs decrease significantly. According with this observation, the oil cleanup was very marginal, and >80% of initially added Bunker C fuel oil was extracted from the anaerobic external sediments after 3 months of oil spill simulation. At the end of experiment, analysis of microbial community of the MSS external sediments revealed the overwhelming dominance of the *Deltaproteobacteria*.

The stimulation of autochthonous bacteria to tackle the pollution in contaminated environment is widely used in the remediation of aerobic sites (Harayama et al., 2004). However, this technology is hardly applicable to oxygen-depleted marine sediments. Additionally, due to high probability of contaminant spreading while removing, the application of *ex-situ* remediation technologies is severely limited. To initiate the self-cleaning process in sediments driven by indigenous aerobic OMHCB, we used *in situ* aeration of polluted anoxic sediments in specially designed MSS. It is generally assumed, that petroleum contamination induced drastic changes in the bacterial community structure associated with a decrease of diversity (Grötzschel et al., 2002; Röling et al., 2002; Yakimov et al., 2005, 2007; Head et al., 2006; Bordenave et al., 2007). These changes were referred to both toxic effect of PHs and a strong selection toward highly specialized hydrocarbon-degrading microorganisms (Harayama et al., 1999; Kasai et al., 2001; Grötzschel et al., 2002; Yakimov et al., 2005, 2007). Accordingly, the most drastic shifts in the MSS bacterial community dynamic was observed at the beginning and after 1 month of the oil spill. As revealed by 16S crDNA clone library analysis, more than a half of MSS microbial population at T_29_, belonged to *Alcanivorax* (43%), *Cycloclasticus* (7%) and *Marinobacter* (5%), the genera of OMHCB, known to play a pivotal role in petroleum degradation in marine environments (Harayama et al., 1999; Röling et al., 2002, 2004; Yakimov et al., 2007). Noteworthy, the dominance of these OMHCB genera was confirmed by both CARD-FISH and qPCR analyses. At the end of the treatment, the level of TERHC degradation in the MSS internal sediments was almost 98%, and the resulting microbial community was characterized by an almost complete extinction of OMHCBs. Both HCA and UniFrac PCoA statistic analyses of the OTUs abundance matrix indicated that the structure of T_90_ microbial community was more similar to initial microbial community structures. As a consequence of successful bioremediation, the *Corophium orientale* eco-toxicological bioassay revealed that toxicity of the MSS internal sediments was substantially lower compared with the untreated external sediments. Thus, to the best of our knowledge, our studies for the first time demonstrated that petroleum-contaminated anaerobic marine sediments could be efficiently recovered by their capping and *in situ* aeration, thus stimulating the self-cleaning potential due to reawakening of residing aerobic OMHCBs.

### Conflict of interest statement

The authors declare that the research was conducted in the absence of any commercial or financial relationships that could be construed as a potential conflict of interest.

## References

[B1] Acosta González A. Rosselló Móra R. Marqués S. (2013). Characterization of the anaerobic microbial community in oil polluted subtidal sediments: aromatic biodegradation potential after the Prestige oil spill. Environ. Microbiol. 15, 77–92 10.1111/j.1462-2920.2012.02782.x 22626032

[B2] Albaigés J. Morales-nin B. Villas F. (2006). The Prestige oil spill: a scientific response. Mar. Pollut. Bull. 53, 205–207 10.1016/j.marpolbul.2006.03.012 16697421

[B3] Altschul S. F. Madden T. L. Schaffer A. A. Zhang J. Zhang Z. Miller W. (1997). Gapped BLAST and PSI-BLAST: a new generation of protein database search programs. Nucleic Acids Res. 25, 3389–3402 10.1093/nar/25.17.3389 9254694 PMC146917

[B4] Amann R. I. Krumholz L. Stahl D. A. (1990). Fluorescent-oligonucleotide probing of whole cells for determinative, phylogenetic, and environmental studies in microbiology. J. Bacteriol. 172, 762–770 1688842 10.1128/jb.172.2.762-770.1990 PMC208504

[B5] Ashelford K. E. Chuzhanova N. A. Fry J. C. Jones A.J. Weightman A. J. (2005). At least 1 in 20 16S rRNA sequence records cur- rently held in public repositories is estimated to contain substantial anomalies. Appl. Environ. Microbiol. 71, 7724–773 10.1128/AEM.71.12.7724-7736.2005 16332745 PMC1317345

[B6] Benton M. J. Malott M. L. Knight S. S. Cooper C. M. Benson W. H. (1995). Influence of sediment composition on apparent toxicity in a solid phase test using bioluminescent bacteria. Environ. Toxicol. Chem. 14, 411–414 10.1002/etc.5620140309

[B7] Bordenave S. Goñi-Urriza M. S. Caumette P. Duran R. (2007). Effects of heavy fuel oil on the bacterial community structure of a pristine microbial mat. Appl. Environ. Microbiol. 73, 6089–6097 10.1128/AEM.01352-07 17704271 PMC2075027

[B8] Bulich A. A. Greene M. W. Underwood S. R. (1992). Measurement of soil and sediment toxicity to bioluminescent bacteria when in direct contact for a fixed time period, in Memorias de Water Environment Federation, 65th Annual Conference and Exposition (Nueva Orleans, Louisiana), 53–64

[B9] Clark R. N. King T. V. V. Klejwa M. Swayze G. Vergo N. (1990). High spectral resolution reflectance spectroscopy of minerals. J. Geophys. Res. 95, 12653–12680 10.1029/JB095iB08p12653

[B10] Clarke K. R. (1993). Non parametric multivariate analyses of changes in community structure. Aust. J. Ecol. 18, 117–143 10.1111/j.1442-9993.1993.tb00438.x19830478

[B11] Clarke K. R. Gorley R. N. (2006). PRIMER v6. User Manual and Tutorial. Plymouth: PRIMER-E

[B12] Cravo-Laureau C. Matheron R. Cayol J. L. Joulian C. Hirschler-Réa A. (2004). *Desulfatibacillum aliphaticivorans* gen. nov., sp. nov., an n-alkane-and n-alkene-degrading, sulfate-reducing bacterium. Int. J. Syst. Evol. Microbiol. 54, 77–83 10.1099/ijs.0.027170-0 14742462

[B13] Dyksterhouse S. E. Gray J. P. Herwig R. P. Lara J. C. Staley J. T. (1995). *Cycloclasticus pugetii* gen. nov., sp. nov., an aromatic hydrocarbon-degrading bacterium from marine sediments. Int. J. Syst. Bacteriol. 45, 116–123 10.1099/00207713-45-1-116 7857792

[B14] Engelen B. Cypionka H. (2009). The subsurface of tidal-flat sediments as a model for the deep biosphere. Ocean Dynam. 59, 385–391 10.1007/s10236-008-0166

[B15] Ferraro G. Bernardini A. David M. Meyer-Roux S. Muellenhoff O. Perkovic M. (2007). Towards an operational use of space imagery for oil pollution monitoring in the Mediterranean basin: a demonstration in the Adriatic Sea. Mar. Pollut. Bull. 54, 403–422 10.1016/j.marpolbul.2006.11.022 17254613

[B16] Gertler C. Yakimov M. M. Malpass M. C. Golyshin P. N. (2010). Shipping-related accidental and deliberate release into the environment, in Handbook of Hydrocarbon and Lipid Microbiology, ed Timmis K. N. (Berlin, Heidelberg: Springer), 243–256 10.1007/978-3-540-77587-4_16

[B17] Golyshin P. N. Chernikova T. N. Abraham W. R. Lünsdorf H. Timmis K. N. Yakimov M. M. (2002). *Oleiphilaceae* fam. nov., to include *Oleiphilus messinensis* gen. nov., sp nov., a novel marine bacterium that obligately utilizes hydrocarbons. Int. J. Syst. Evol. Microbiol. 52, 901–911 10.1099/ijs.0.01890-0 12054256

[B18] Good I. J. (1953). The population frequencies of species and the estimation of population parameters. Biometrika. 40, 237–264 10.2307/2333344

[B15a] Gray S. B. Classen A. T. Kardol P. Yermakov Z. Miller R. M. (2011). Multiple climate change factors interact to alter soil microbial community structure in an old-field ecosystem. Soil Sci. Soc. Am. J. 75, 2217–2226 10.2136/sssaj2011.013520023089

[B19] Grötzschel S. Köster J. Abed R. M. M. De Beer D. (2002). Degradation of petroleum model compounds immobilized on clay by a hypersaline microbial mat. Biodegradation 13, 273–283 10.1023/A:1021263009377 12521291

[B20] Hammer Ø. Harper D. A. T. Ryan P. D. (2001). PAST: palaeontological statistics software package for education and data analysis. Palaeontol. Electron. 4, 9.

[B21] Hara A. Syutsubo K. Harayama S. (2003). *Alcanivorax* which prevails in oil-contaminated seawater exhibits broad substrate specificity for alkane degradation. Environ. Microbiol. 5, 746–753 10.1046/j.1468-2920.2003.00468.x 12919410

[B22] Harayama S. Kasai Y. Hara A. (2004). Microbial communities in oil-contaminated seawater. Curr. Opin. Biotechnol. 15, 205–214 10.1016/jcopbio.2004.04.002 15193328

[B23] Harayama S. Kishira H. Kasai Y. Shutsubo K. (1999). Petroleum biodegradation in marine environments. J. Mol. Microbiol. Biotechnol. 1, 63–70 10941786

[B24] Head I. M Jones D. M Röling W. F. (2006). Marine microorganisms make a meal of oil. Nat. Rev. Microbiol. 4, 173–182 10.1038/nrmicro1348 16489346

[B24a] ISPRA (2013). Batterie di saggi ecotossicologici per sedimenti e acque interne, in Manuali di Ecotossicologia, eds ISPRA-settore editoria ISBN: 978-88-448-0607-1.

[B25] Karner M. B. Fuhrman J. (1997). Determination of active marine bacterioplankton: a comparison of universal 16S rRNA probes, autoradiography, and nucleoid staining. Appl. Environ. Microbiol. 63, 1208–1213 16535563 10.1128/aem.63.4.1208-1213.1997 PMC1389541

[B26] Kasai Y. Kishira H. Sasaki T. Syutsubo K. Watanabe K. Harayama S. (2002). Predominant growth of *Alcanivorax* strains in oil contaminated and nutrient supplemented seawater. Environ. Microbiol. 4, 141–147 10.1046/j.1462-2920.2002.00275.x 12000314

[B27] Kasai Y. Kishira H. Syutsubo K. Harayama S. (2001). Molecular detection of marine bacterial populations on beaches contaminated by the Nakhodka tanker oil spill accident. Environ. Microbiol. 3, 246–255 10.1046/j.1462-2920.2001.00185.x 11359510

[B28] Kemp P. F. Aller J. Y. (2004). Bacterial diversity in aquatic and other environments: what 16S rDNA libraries can tell us. FEMS Microbiol. Ecol. 47, 161–177 10.1016/S0168-6496(03)00257-5 19712332

[B28a] Kuwae T. Hosokawa Y. (1999). Determination of the abundance and biovolume of bacteria in sediments by dual staining with 4′,6-diamidino-2-phenylindole and acridine orange: relationship to dispersion treatment and sediment characteristics. Appl. Environ. Microbiol. 65, 3407–3412 10427027 10.1128/aem.65.8.3407-3412.1999 PMC91512

[B29] Lane D. J. (1991). 16/23S rRNA sequencing, in Nucleic Acid Techniques in Bacterial Systematics, eds Stackerbrandt E. Goodfellow M. (New York, NY: Wiley), 115–175

[B30] Lozupone C. A. Hamady M. Kelley S. T. Knight R. (2007). Quantitative and qualitative β diversity measures lead to different insights into factors that structure microbial communities. Appl. Environ. Microbiol. 73, 1576–1585 10.1128/AEM.01996-06 17220268 PMC1828774

[B31] Ludwig W. Strunk O. Westram R. Richter L. Meier H. Yadhukumar (2004). ARB: a software environment for sequence data. Nucleic Acids Res. 32, 1363–1371 10.1093/nar/gkh293 14985472 PMC390282

[B32] McKew B.A. Coulon F. Yakimov M. M. Denaro R. Genovese M. Smith C. J. (2007). Efficacy of intervention strategies for bioremediation of crude oil in marine systems and effects on indigenous hydrocarbonoclastic bacteria. Environ. Microbiol. 9, 1562–1571 10.1111/j.1462-2920.2007.01277.x 17504493

[B33] Microtox System Operating Manual (1982). Microbics Operations. Carlsbad, CA: Beckman Instruments, Inc

[B34] Onorati F. Bigongiari N. Pellegrini D. Giuliani S. (1999). The suitability of *Corophium orientale* (Crustacea, Amphipoda) in harbour sediment toxicity bioassessment. Aquat. Ecosys. Health Manage. 2, 465–473 10.1016/S1463-4988(99)00030-5

[B35] Païssé S. Coulon F. Goñi-Urriza M. Peperzak L. McGenity T. J. Duran R. (2008). Structure of bacterial communities along a hydrocarbon contamination gradient in a coastal sediment. FEMS Microbiol. Ecol. 66, 295–305 10.1111/j.1574-6941.2008.00589 18803671

[B36] Païssé S. Goñi-Urriza M. Coulon F. Duran R. (2010). How a bacterial community originating from a contaminated coastal sediment responds to an oil input. Microb. Ecol. 60, 394–405 10.1007/s00248-010-9721-7 20652237

[B37] Pernthaler A. Pernthaler J. Amann R. (2002). Fluorescence *in situ* hybridization and catalyzed reporter deposition for the identification of marine bacteria. Appl. Environ. Microbiol. 68, 3094–3101 10.1128/AEM.68.6.3094-3101.2002 12039771 PMC123953

[B38] Pruesse E. Quast C. Knittel K. Fuchs B. M. Ludwig W. G. Peplies J. (2007). SILVA: a comprehensive online resource for quality checked and aligned ribosomal RNA sequence data compatible with ARB. Nucleic Acids Res. 35, 7188–7196 10.1093/nar/gkm864 17947321 PMC2175337

[B39] Psarros G. Skjong R. Eide M. S. (2010). Under-reporting of maritime accidents. Accid. Anal. Prev. 42, 619–625 10.1016/j.aap.2009.10.008 20159087

[B40] Rasmussen R. (2001). Quantification on the LightCycler, in Rapid Cycle Real-Time PCR, Methods and Applications, eds Meuer S. Wittwer C. Nakagawara K. (Heidelberg: Springer Press), 21–34 10.1007/978-3-642-59524-0_3

[B41] Ringwood A. H. DeLorenzo M. E. Ross P. E. Holland A. F. (1997). Interpretation of Microtox® solid phase toxicity tests: the effects of sediment composition. Environ. Toxicol. Chem. 16, 1135–1140 10.1002/etc.5620160607

[B42] Rocchetti L. Beolchini F. Ciani M. Dell'Anno A. (2011). Improvement of bioremediation performance for the degradation of petroleum hydrocarbons in contaminated sediments. Appl. Environ. Soil Sci. 2011:319657 10.1155/2011/319657

[B43] Röling W. F. Milner M. G. Jones D. M. Fratepietro F. Swannell R. P. Daniel F. (2004). Bacterial community dynamics and hydrocarbon degradation during a field-scale evaluation of bioremediation on a mudflat beach contaminated with buried oil. Appl. Environ. Microbiol. 70, 2603–2613 10.1128/AEM.70.5.2603-2613.2004 15128509 PMC404410

[B44] Röling W. F. Milner M. G. Jones D. M. Lee K. Daniel F. Swannell R. P. (2002). Robust hydrocarbon degradation and dynamics of bacterial communities during nutrient-enhanced oil spill bioremediation. Appl. Environ. Microbiol. 68, 5537–5548 10.1128/AEM.68.11.5537-5548.2002 12406747 PMC129918

[B45] Rosselló-Mora R. Amann R. (2001). The species concept for prokaryotes. FEMS Microbiol. Rev. 25, 39–67 10.1016/S0168-6445(00)00040-1 11152940

[B46] Roussel E. G. Sauvadet A. L Allard J. Chaduteau C. Richard P. Cambon Bonavita M. A. (2009). Active archaeal methane cycling communities associated with gassy subsurface sediments of marennes-oléron bay (France) Science 320, 1046 10.1126/science.1154545 18497290

[B47] Schloss P. D. Handelsman J. (2005). Introducing DOTUR, a computer program for defining operational taxonomic units and estimating species richness. Appl. Environ. Microbiol. 71, 1501–1506 10.1128/AEM.71.3.1501-1506.2005 15746353 PMC1065144

[B48] Syutsubo K. Sinthurat N. Ohashi A. Harada H. (2001). Population dynamics of anaerobic microbial consortia in thermophilic granular sludge in response to feed composition change. Water Sci. Technol. 43, 59–66 11379113

[B50] Widdel F. Rabus R. (2001). Anaerobic biodegradation of saturated and aromatic hydrocarbons. Curr. Opin. Biotech. 12, 259–276 10.1016/S0958-1669(00)00209 11404104

[B51] Wilkes H. Vieth A. Elias R. (2008). Constraints on the quantitative assessment of in-reservoir biodegradation using compound-specific stable carbon isotopes. Org. Geochem. 39, 1215–1221 10.1016/j.orggeochem.2008.02.013

[B52] Yakimov M. M. Denaro R. Genovese M. Cappello S. D'Auria G. Chernikova T. N. (2005). Natural microbial diversity in superficial sediments of Milazzo Harbor (Sicily) and community successions during microcosm enrichment with various hydrocarbons. Environ. Microbiol. 7, 1426–1441 10.1111/j.1462-5822.2005.00829.x 16104865

[B53] Yakimov M. M. Giuliano L. Denaro R. Crisafi E. Chernikova T. N. Abraham W. R. (2004). *Thalassolituus oleivorans* gen. nov., sp. nov., a novel marine bacterium that obligately utilizes hydrocarbons. Int. J. Syst. Evol. Microbiol. 54, 141–148 10.1099/ijs.0.02424-0 14742471

[B54] Yakimov M. M. Giuliano L. Gentile G. Crisafi E. Chernikova T. N. Abraham W. R. (2003). *Oleispira antarctica* gen. nov., sp. nov., a novel hydrocarbonoclastic marine bacterium isolated from Antarctic cosatal sea water. Int. J. Syst. Evol. Microbiol. 53, 779–785 10.1099/ijs.0.02366-0 12807200

[B55] Yakimov M. M. Golyshin P. N. Lang S. Moore E. R. B. Abraham W. R. Lünsdorf H. (1998). *Alcanivorax borkumensis* gen. nov., sp. nov., a new, hydrocarbon-degrading and surfactant-producing marine bacterium. Int. J. Syst. Bacteriol. 48, 339–348 10.1099/00207713-48-2-339 9731272

[B56] Yakimov M. M. Timmis K. N. Golyshin P. N. (2007). Obligate oil-degrading marine bacteria. Curr. Opin. Biotech. 18, 257–266 10.1016/j.copbio.2007.04.006 17493798

